# Effects of foliar-sprayed bio-fertilizer with N-fixing *Methylobacterium symbioticum* on morpho-physiological traits of maize under varying N fertilization rates

**DOI:** 10.3389/fpls.2025.1661290

**Published:** 2025-09-04

**Authors:** Pranay Kumar Bolla, Anna Panozzo, Edoardo Minozzi, Francesco Valente, Silvia Potestio, Giovanna Visioli, Isabel Martinez-Sañudo, Teofilo Vamerali

**Affiliations:** ^1^ Department of Agronomy, Food, Natural Resources, Animals and the Environment, University of Padua, Legnaro, Italy; ^2^ Department of Chemistry, Life Sciences and Environmental Sustainability, University of Parma, Parma, Italy

**Keywords:** ACC deaminase, nitrogen fertilization, grain yield and quality, leaf stay-green, root electrical capacitance, plant growth promoting bacteria (PGPB)

## Abstract

Nitrogen (N) fertilization remains a critical challenge in sustainable agriculture. Plant growth-promoting bacteria offer a promising strategy to enhance nitrogen use efficiency and improve cereal crop productivity while reducing reliance on synthetic inputs. This open-field study evaluated the morpho-physiological effects of foliar application of *Methylobacterium symbioticum* (MS) on maize in NE Italy. Following a baseline application of liquid digestate (300 kg N ha^-^¹) to all plots, four treatments were compared: N300 (digestate only, serving as the control); N300 + MS (digestate with MS); N350 (digestate with 50 kg ha^-^¹ chemical N); and N320 + MS (digestate with 20 kg ha^-^¹ chemical N and MS). ESEM imaging confirmed colonization of leaf surfaces by *M. symbioticum*. Its application significantly promoted aboveground growth and delayed leaf senescence by improving chlorophyll retention, increasing seasonal average SPAD from 46.9 in control to 49.4 (+5.3%, N300 + MS) and 48.8 (+4.1%, N320 + MS), likely mediated by the ascertained ACC-deaminase activity of MS. Root electrical capacitance showed treatment-specific effects, with the highest readings under the N320 + MS treatment (+54% vs. control at flowering). Yield responses were non-linear with respect to N dose, with N300 + MS showing a 12% (1,364 g/m²) and N320 + MS a 6% non-significant increase vs. control. MS-treated plants also exhibited a non-significant 5% increase in grain protein content, but significantly higher aboveground N accumulation. It is concluded that, this microbial inoculation strategy can enhance N use efficiency, particularly under reduced synthetic fertilization, presenting an environmentally-friendly and sustainable agricultural strategy for maize cultivation.

## 1 Introduction

Maize (*Zea mays* L.) is a pivotal crop globally, crucial for food security, economic growth, and industry. It supplies over 30% of the dietary calories for roughly 4.5 billion people in developing countries (Shiferaw et al., 2011), while also serving as a primary source of livestock feed and industrial materials. Its cultivation under various climatic conditions further highlights its global significance, especially under a climate change scenario (Kumar and Bhatt, 2012). Maize growth critically depends on an adequate N supply, as its deficiency leads to stunted growth and reduced yields (Guo et al., 2023; Kafle et al., 2023). Intensive agricultural practices have progressively reduced soil organic matter and N levels, leading to widespread reliance on synthetic fertilizers. While chemical fertilizers effectively enhance crop productivity, they are linked to environmental issues such as nitrate leaching and increased greenhouse gas emissions (Galloway et al., 2008). As a result, there is growing interest in sustainable farming methods that minimize chemical inputs while maintaining high maize productivity.

The application of biostimulants, especially microbial inoculants like N-fixing bacteria, presents a promising alternative to chemical fertilizers in agriculture (Rouphael and Colla, 2020). Unlike leguminous plants, maize is unable to establish a symbiotic relationship with N-fixing bacteria, although it can form associations with various rhizosphere bacteria and endophytes that are capable of providing various benefits, including N-fixation (Pankievicz et al., 2019). Biostimulants, which include a variety of compounds and microorganisms, can enhance plant growth and tolerance to stress in addition to minimal direct nutrient supply. Among plant aiding bacteria, *Azospirillum*, particularly *A. brasilense*, develops favourable root associations in maize, enhancing N use efficiency, improving root and shoot growth, and increasing yields by up to 30% under N-limited conditions (Barbosa et al., 2022; Teodoro-Cerna et al., 2020). Similarly, *Bacillus* and *Pseudomonas* can improve maize performance through mechanisms such as phosphate solubilization, siderophore production, phytohormone synthesis, and biofilm formation (Vieira Velloso et al., 2020). Some bacteria, such as *Bacillus subtilis*, can also improve drought tolerance by thickening roots, altering root structure and increasing plant biomass (Vardharajula et al., 2011).

Inoculation through the root zone has long been conventionally utilized to harness plant-microbe interactions, but it frequently struggles with severe microbial competition in the soil and uneven colonization of root surfaces, although seed treatments have often provided positive results (Francioli et al., 2025; Wang and Kuzyakov, 2024). In contrast, foliar spraying of microbial inoculants bypasses soil microbial competition, allowing for faster leaf surface colonization. This application method enables both epiphytic and endophytic beneficial bacteria to interact directly with leaf-level physiology, modulating processes such as photosynthesis, stomatal conductance, and antioxidant production (Preininger et al., 2018).

Among biological tools that can contribute to improve plant health and productivity, *Methylobacterium symbioticum* a novel species isolated from spores of the symbiotic fungus *Glomus iranicum* var. *tenuihypharum*, stands out as a notable N-fixing bacterium recently providing an advantage for maize cultivation although with varying outcomes (Pascual et al., 2020; Rodrigues et al., 2024). *Methylobacterium* species are found in a wide range of environments, such as soil, water, leaf surfaces, plant nodules, grains, and the atmosphere, exhibiting considerable versatility (Wellner et al., 2012), having been identified in over 70 plant species. Notably, *Methylobacterium nodulans* has been shown to induce root nodules in *Crotalaria* leguminous plants (Jourand et al., 2004), while a majority of *Methylobacterium* species form either epiphytic or endophytic relationships with host plants. Epiphytic strains can enter through leaf surfaces and infiltrate stomatal openings, forming endophytic communities that positively affect the host plant (Ardanov et al., 2012; Dourado et al., 2015; Kutschera, 2007). Approximately 30% of *Methylobacterium* species found in the phyllosphere are capable of fixing N, converting atmospheric N_2_ into ammonium to support their own growth and development, with possible release of N to the host plant (Madhaiyan et al., 2015). *Methylobacterium symbioticum* have been demonstrated to potentially reduce N chemical fertilization in various crops, such as maize, rice, wheat, and wine grape. Recent research supports the potential of *Methylobacterium symbioticum* as a sustainable microbial inoculant capable of boosting nitrogen use efficiency (NUE) and enhancing crop yields through foliar application. The studies by Torres Vera et al. (2024), confirmed that foliar application of *M. symbioticum* significantly improves maize performance by enhancing N uptake while reducing the need for high levels of external N fertilizers. Similar positive effects have also been observed in strawberries, where the bacterium enhanced growth and yield even under low N availability (Silva et al., 2022).

However, the effectiveness of *M. symbioticum* is not consistent across all crop species or environmental conditions. While positive effects have been observed in maize and strawberries, studies on lettuce have shown minimal improvements in growth and N nutrition (Arrobas et al., 2024). This variability highlights the importance of crop- or even variety-specific evaluations when considering the use of this inoculant. In contrast, crops like wheat and sugarcane have demonstrated clear benefits, especially under N-limited conditions (Jirakkakul et al., 2023; Valente et al., 2024), further emphasizing the potential for targeted agronomic applications. Studies by Knief et al. (2010), emphasized that environmental conditions have a stronger influence on the composition of *Methylobacterium* communities in the phyllosphere than the plant species itself. For this reason, it is essential to evaluate the agronomic performances of *M. symbioticum* across various environmental conditions, particularly, regarding the foliar application of this bacterium on maize under different N fertilization regimes.

Building on this existing body of research, which highlighted the potential of microbial inoculants to enhance NUE and crop productivity, this study aimed to carry out a comprehensive assessment of the agronomic impacts of *M. symbioticum* applied on maize through foliar spraying. The objectives were thematically structured as follows: *i*) microbial colonization and mechanism: determine the efficiency of leaf colonization by sprayed *M. symbioticum* and assess its ACC-deaminase activity; *ii)* Plant physiological responses: quantify maize response following foliar-sprayed bacteria by measuring morpho-physiological characteristics, such as growth indices (plant height, leaf number, above-ground biomass), leaf area index (LAI), and chlorophyll content (as SPAD measurement) to assess possible enhancements in plant vigor and delayed senescence; *iii*) Root development and yield performance: analyze root growth response using indirect non-destructive measurements of root electrical capacitance, and yield components such as grain yield and kernel weight, along with grain quality attributes including moisture, protein, oil, and starch content at harvest.

Recognizing that N availability is a critical determinant in maize productivity, this study investigated the interaction between varying levels of N fertilization and the application of *M. symbioticum* to determine if the bacterium improves nitrogen use efficiency and potentially compensate for reduced chemical N input.

## 2 Materials and methods

### 2.1 Site description and experimental design

The field trial was conducted during 2023 growing season at the Minozzi farm, located in Legnago, Verona, NE Italy (8 m a.s.l.). Based on the contents of silt (54.2%), clay (29.4%) and sand (16.4%), the soil was classified as silty-clay-loam (USDA), with a pH of 6.9, with very high levels of both organic matter content (16.8%) and cation exchange capacity (CEC of 52.3 cmol (+) kg^-1^), and total nitrogen (N) content (6.41 g kg^-1^) before sowing.

Climatic data were collected from the meteorological station in Legnago-Vangadizza, 2 km from the experimental site (ARPAV - weather service center of the Veneto region). The preceding crop was durum wheat, grown in the 2021–2022 season, subsequently leaving the land fallow until spring 2023.

The experiment aimed to evaluate the impact of the N-fixing bacterium *Methylobacterium symbioticum* strain SB23 provided through the commercial product BlueN^®^ (Corteva Agriscience, Cremona - IT) in combination with different doses of chemical N fertilizer as top-dress application (**Table 1**). This bacterium is expected to provide from at least 30 up to 50 kg of N ha^-^¹ to the inoculated plants through N-fixation, as a theoretical estimate.

**Table 1:** Overview of fertilization strategy and biofertilizer applications for each treatment in the study.

Treatment name	Organic N from digestate (kg ha^-1^)	Top-dress chemical N fertilizer (kg ha^-1^)	Total N provided (kg ha^-1^)	*Methylobacterium symbioticum* inoculation
N300	300	0	300	NO
N300 + MS	300	0	300	YES
N350	300	50	350	NO
N320 + MS	300	20	320	YES

Total N provided’ values refer to organic N supplied from the digestate and chemical fertilizer combined, excluding any contribution from *M. symbioticum*.

In February 2023, 75 t ha^-1^ of liquid digestate (from green wastes) containing 0.4% N, equivalent to 300 kg N ha^-1^, was applied in the whole experimental field, followed by plowing to a depth of 30 cm. The seedbed was prepared using a rotary harrow, and maize sowing took place on 3 April. Weed control occurred in post-emergence on 2 May at the 4-5-leaf stage of maize, using the active ingredients (a.i.) Nicosulfuron and Rimsulfuron against monocot and the a.i. Mesotrione against dicot species.

Top-dress N fertilization was applied on 9 June with ammonium sulfate (containing 21% N and 24% S) as granular fertilizer, followed by burial through hoeing; the rate of chemical N, with or without the *Methylobacterium symbioticum* biofertilizer (MS), allowed for the comparison of four treatments (**Table 1**). Treatments were named as follows: N300 (digestate only, serving as the control); N300 + MS (digestate with MS); N350 (digestate with 50 kg ha^-^¹ chemical N); and N320 + MS (digestate with 20 kg ha^-^¹ chemical N and MS).

*M. symbioticum*, available as wettable powder (3 × 10^7^ CFU g^−1^), was mechanically sprayed on maize on 12 June (8-10-leaf stage) at a dose of 333 g ha^-1^ with 300 L ha^-1^ of water. The bacterium is recommended to be applied at the 6-8-leaf stage, but in this study, it was applied somewhat later due to continuous rainfall during May.

The trial was carried out in a 4-hectare field area, cultivating the short-season (FAO class 300) maize hybrid P9241 (Pioneer – Corteva Agriscience), with a sowing density of 8 plants m^-2^ (75 cm interrow × 16.6 cm on the row). The field was divided into four strips, each comprising three plots/replicates (n = 3). Within each plot, two individual plants were designated as sub-replicates for SPAD and root capacitance measurements. In addition, 8 and 6 plants per plot were assessed for shoot parameters and yield response, respectively. To mitigate spatial heterogeneity and ensure statistical robustness, all treatments followed a randomized complete block design. Each plot, measuring 4 m in length and 3 m in width (4 maize rows), was located centrally within the treated zone. The crop was harvested on 28 August 2023, approximately two weeks after the final survey date.

### 2.2 Environmental scanning electron microscope imaging of bacteria–leaf interactions

ESEM was employed to assess the colonization ability of *M. symbioticum*-based inoculum on maize leaves. This technique facilitates the analysis of *in-vivo* biological specimens without histological processing, enabling direct visualization of bacterial colonization on the leaf surface, thus providing an accurate representation of the plant-bacteria interaction (Visioli et al., 2014). To prepare the bacterial inoculum, a suspension of *M. symbioticum* was plated on Luria–Bertani (LB) solid medium at various dilutions, to isolate single colonies, and incubated at 28°C for 72 hours. Single isolated colonies were cultured in 100 mL of LB medium on a rotary shaker at 28°C for 48 h. Following centrifugation, bacterial cells were re-suspended in sterile water to reach a final inoculum density of 3 × 10^7^ CFU mL^-^¹.

For the inoculation experiment, maize plants were grown under sterile conditions within a climatic chamber on ½ MS agar medium supplemented with 1% sucrose. At the 3-leaf stage, a bacterial suspension of *M. symbioticum* was applied to the leaf surfaces. Inoculated plants were allowed to grow for additional 7 days, while non-inoculated plants served as controls. At the end of the incubation period, fresh leaf sections of 5-mm diameter were excised with a sterile lancet. Specimens were mounted on an ESEM sample holder, ensuring minimal dehydration to preserve structural integrity. Morphological analysis through imaging was performed using Quanta™ 250 FEG Environmental Scanning Electron Microscope (ESEM) (FEI, Hillsboro, OR, USA) in wet mode: all micrographs were taken using a gaseous secondary electron detector at an accelerating voltage of 10 kV, while the chamber relative humidity was initially set at 100%, then progressively reduced to 80% and maintained at that level throughout the imaging process (Visioli et al., 2014).

### 2.3 ACC deaminase activity assay in *Methylobacterium symbioticum*

The strain SB23 of *Methylobacterium symbioticum* was assessed for 1-aminocyclopropane-1-carboxylate (ACC) deaminase activity using a qualitative assay, where its growth was compared on two distinct media, i.e., one with ACC as the sole N source, and the other without any N source (Penrose and Glick, 2003). The bacterium was cultured overnight in 10 mL of LB liquid medium, agitated at 130 rpm at 28°C. Bacterial abundance was evaluated by optical density (OD) at 600 nm wavelength measured using a Varian Cary 50 UV-Visible spectrophotometer. The bacterial culture was then diluted to 10^8^ cells mL^-1^ with sterile double-distilled water and spread onto LB agar medium (Singh et al., 2022). After 24 hours of incubation at 28°C, a bacterial colony was transferred to a Petri dish containing modified DF minimal salts medium, which included glucose (4.0 g/L), citric acid (2.0 g/L), potassium dihydrogen phosphate (KH_2_PO_4_ 4.0 g/L), disodium hydrogen phosphate (Na_2_HPO_4_ 6.0 g/L), magnesium sulfate heptahydrate (MgSO_4_·7H_2_O, 0.2 g/L), and a micronutrient solution (comprising ferrous sulfate (FeSO_4_ 100 mg/100 mL), boric acid (H_3_BO_3_, 10 mg/100 mL), zinc sulfate heptahydrate (ZnSO_4_·7H_2_O, 124.6 mg/100 mL), sodium molybdate (Na_2_MoO_4_ 10 mg/100 mL), copper sulfate (CuSO_4_ 78.22 mg/100 mL), and manganese sulfate (MnSO_4_ 11.19 mg/100 mL). Additionally, 3 mM ACC served as the sole N source (Singh et al., 2022). As a negative control, the DF minimal medium was prepared without ACC. The ability of the bacterial isolates to grow on DF medium supplemented with ACC was evaluated after 72 hours of incubation at 28°C and compared to the negative controls. *Bosea robiniae* and *Pseudomonas umsongensis* were used as negative and positive ACC deaminase-producing references, respectively (Bertola et al., 2019). Bacterial colonization in the DF medium was recorded as colored bands, i.e., pinkish-orange for *M. symbioticum* and light yellow for *P. umsongensis*.

### 2.4 Shoot parameters

During maize growth, the leaf chlorophyll content was indirectly assessed through SPAD (Soil and Plant Analysis Development) measurements. Data were collected from two randomly selected plants in each plot/replicate of each treatment, using a SPAD 502 chlorophyll meter (Konica-Minolta, Hong Kong). For each plant, SPAD readings were taken on every fully expanded leaf, accounting for its position along the plant height. The SPAD value for each leaf was calculated as the average of three readings taken along the length of the leaf blade (at one-quarter, one-half, and three-quarters). Measurements were conducted on the same plants at intervals of 2–3 weeks on the following dates: 23 June (flowering stage), 5 July (milking stage), 25 July (dough stage), and 5 August (early physiological maturity stage).

On 19 July, at late milk stage, 8 randomly selected plants per plot were also harvested by cutting them at the base to analyze aboveground biomass. Morphological parameters measured included plant height, number of leaves and number of ears per plant. After stem, leaf, and ear separation, leaf area index (LAI) was calculated for each plot/replicate using the LI-3100C Area Meter (Li-Cor instruments, Lincoln, NE, USA). Fresh weights were measured for each fraction, and dry weights obtained after oven-drying at 110°C for 72 h.

### 2.5 Root analysis

The root electrical capacitance, as an index of root mass/activity (Cseresnyés et al., 2018), was measured non-destructively using a digital LCR multimeter (Escort ELC 131-D, Taipei, Taiwan) at 120 Hz frequency, and the values were reported in nanofarads (nF). At the beginning of the experiment, 60-cm long stainless-steel electrodes were inserted into the soil to serve as negative electrodes, with one per plot/replicate of the four treatments. Each electrode was positioned along the sowing row between two plants, allowing them to record 2 measurements (one for each neighboring plant) per plot, this setup resulting in a total of 6 measurements per plot. According to the methodology by Streda et al. (2020), the negative electrode was placed approximately 8 cm from the base of the two plants and inserted to a depth of 55 cm, presumably covering the 0–55 cm soil depth range during root capacitance measurements. A clamp was equipped with a 1-mm thick needle that acted as the positive electrode and was inserted 2 mm into the stem base at a height of 5 cm during electrical capacitance measurements. Measurements were conducted on the same dates as SPAD index measurements. The recorded values were taken five seconds after connecting the electrodes to ensure stability of the readings.

### 2.6 Yield and grain quality

After reaching physiological maturity, with an average grain moisture content of about 19%, 6 plants per plot were harvested by cutting their stems just above ground level. For each sample, the stems were manually separated from the ears, and the kernels were then threshed from the corncob. Two fractions, i.e., kernels and crop residues (including stems, bracts and corncobs of ears) were oven-dried at 65°C for 72 h to determine dry weight. In addition to grain yield, the harvest index (HI, i.e., grain-to-total shoot DW ratio) and the thousand seed weight (TSW) were determined. For the latter, 3 sub-plots of 1,000 seeds from each plot/replicate were weighed and averaged, using the Contador seed counter (Pfeuffer, Kitzingen, Germany).

Grain quality was assessed using Near Infrared Spectroscopy (NIRS) technology by analysing a sample of approximately 300 g from each plot/replicate with an Infratec - 1241 instrument (Foss Analytical, Hillerod, Denmark). The quality parameters assessed were moisture, oil, and starch content, expressed as a percentage value on DW. Additionally, the protein content of grains and crop residues was measured using the Kjeldal method (Kirk, 1950): the obtained nitrogen content (%) was multiplied by the crop-specific nitrogen-to-protein conversion factors of 6.25.

### 2.7 Elemental composition in grain and crop residue

Elemental analysis of maize grains and crop residues was conducted to measure the concentrations of Ca, Cu, Fe, K, Mg, Mn, Na, P, S, and Zn. Samples were dehydrated, pulverized, and subjected to acid digestion before analysis. The element concentrations were quantified in mg kg^–1^ DW using inductively coupled plasma optical emission spectrometry (ICP-OES), adhering to standardized procedures for accuracy and precision (Khan et al., 2022).

### 2.8 Statistical analysis

The data for all assessed parameters were subjected to analysis of variance (ANOVA) using Statgraphics ver. 19 software. Residual were examined leveraging the software’s internal diagnostic tools, residuals were evaluated via Q–Q plots for normality and residuals vs. fitted values for homogeneity of variance, and no significant deviations were observed. Treatment means were separated by Tukey’s test when significant differences were detected, using a significance level of p ≤ 0.05 used for all comparison.

Pearson’s correlation coefficients (r) were computed to assess the relationships between morphological, physiological, and yield parameters using CoStat software (version 6.204, CoHort Software, Monterey, CA, USA). Correlations were deemed statistically significant at p ≤ 0.05 (*), p ≤ 0.01 (**), and p ≤ 0.001 (***). A correlation matrix was generated to illustrate the relationships between seasonal SPAD values, root capacitance during early growth stage, morphological traits (plant height, leaf count, LAI), biomass components (total shoot biomass, ear count), yield metrics (grain yield, grain moisture, 1000-seed weight, harvest index), and grain quality attributes (protein, oil, and starch content).

## 3 Results

### 3.1 Weather conditions during the trial

Precipitation patterns revealed significant monthly variation throughout the trial period (April-August 2023; **Supplementary Figure S1**). While April, June and July recorded precipitation levels (~40 mm) slightly lower than the historical means, abundant rainfall occurred in May and August (**Supplementary Figure S2**). May notably experienced a peak precipitation of 173 mm, significantly exceeding the 2012–2022 decadal average of 93 mm. Notably, the 48-hour window surrounding 12 June during foliar spraying of *M. symbioticum* was characterized by the absence of precipitation events.

Regarding thermal conditions, average daily temperatures were generally aligned with historical data, with some deviations. In April, temperatures registered approximately 1.2°C below the decadal average, potentially impacting plant establishment in early stages. Conversely, July exhibited a notable increase in temperature, with values of 1.0°C above the average, potentially accelerating plant’s growth and ripening during this time (**Supplementary Figure S3**). The period between 25 July and 5 August saw consistently high temperatures, potentially causing heat stress, but with plants approaching senescence.

### 3.2 Leaf bacterial colonization

**Figure 1:**
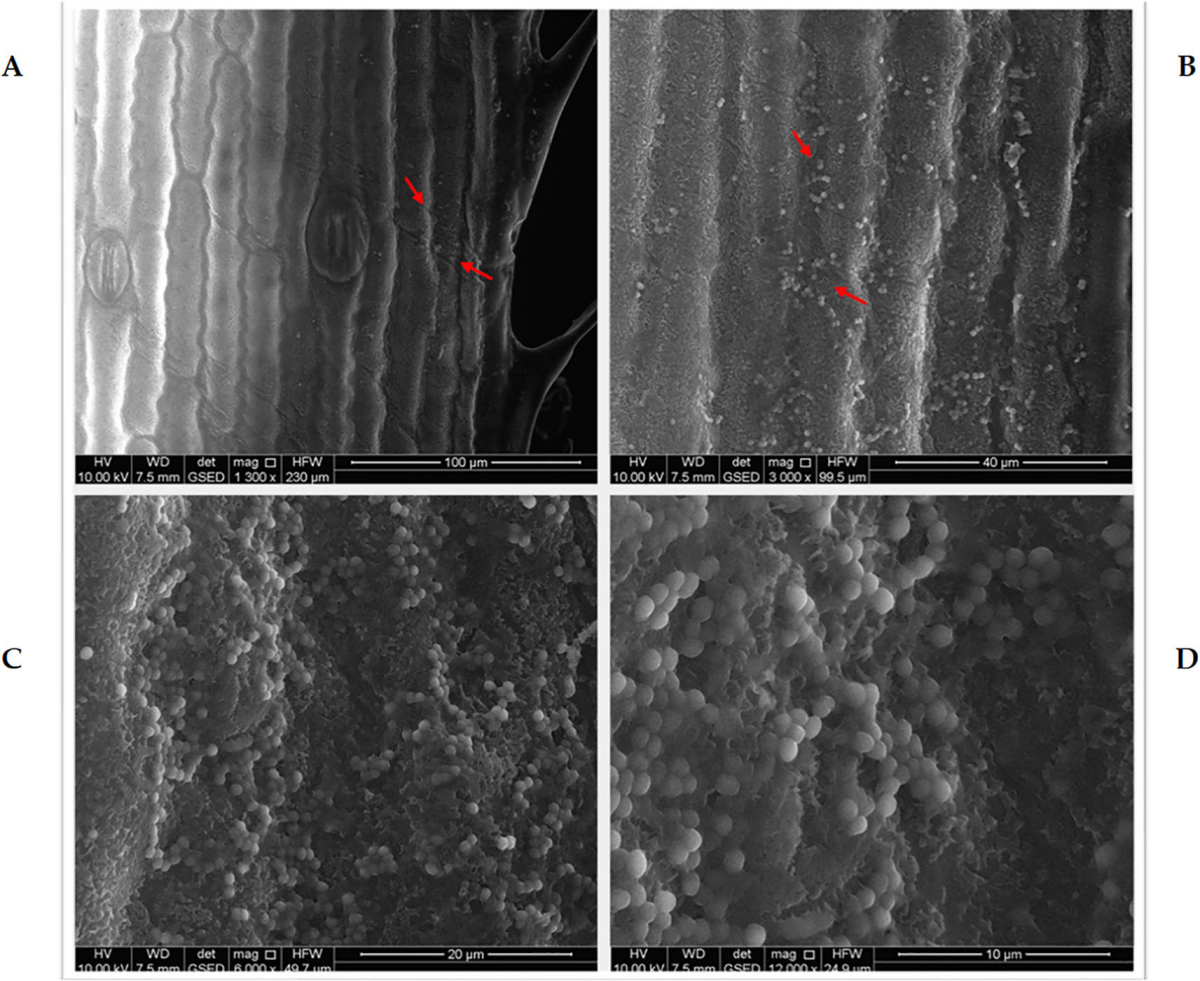
Environmental scanning electron microscopy (ESEM) micrographs illustrating the colonization of *in-vitro* maize leaf surfaces by *Methylobacterium symbioticum* strain SB23 under aseptic conditions. Images depict increasing magnifications: 1300× **(A)**, 3000× **(B)**, 6000× **(C)**, and 12000× **(D)**. Red arrows in **(A)** indicate the region of interest on the leaf surface that is further magnified in **(B–D)** to enhance visualization of bacterial attachment and biofilm formation.

Environmental scanning electron microscopy (ESEM) analysis confirmed successful colonization of *M. symbioticum* on leaves of maize plantlets subjected to foliar spraying under aseptic conditions. The micrographs (**Figure 1**) demonstrate qualitative and predominant localization of bacterial populations along foliar vascular structures and stomatal regions, with sequential magnification revealing distinct bacterial aggregations. Micrographs 2A through 2D present colonization gradients at increasing magnifications ranging from 1,300× to 12,000×. Bacterial cells appear as distinctive spherical formations with adherence to the epidermal surfaces, providing evidence of effective colonization, and their potential to enter through stomal openings. On the contrary, examination of non-inoculated control specimens cultivated under aseptic conditions, as expected, revealed no bacterial presence on foliar surfaces, thereby confirming the specificity of colonization attributable to the bacterial inoculation.

### 3.3 ACC deaminase activity of *Methylobacterium symbioticum*

**Figure 2:**
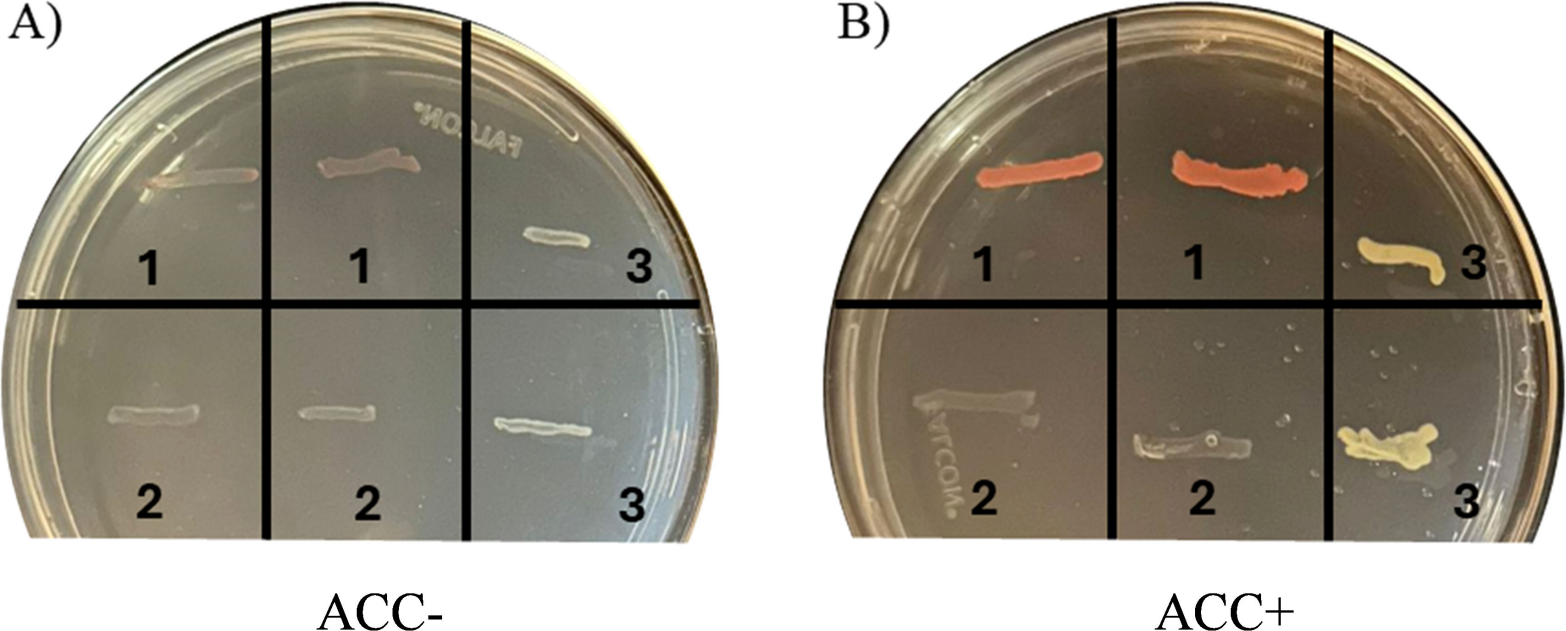
Qualitative test for 1-aminocyclopropane-1-carboxylic acid (ACC) deaminase activity. **(A)** DF minimal medium without any N source; **(B)** DF minimal medium with ACC as a sole N source. 1. *Methylobacterium symbioticum* SB23; 2. Negative control: *Bosea robiniae*; 3. Positive control: *Pseudomonas umsongensis*. Note colonization of *M. symbioticum* SB23 and *P. umsongensis* (coloured bands) when only ACC was present in the growing medium.

*Methylobacterium symbioticum* SB23 demonstrated to possess the ACC deaminase activity, as evidenced by its growth on DF medium supplemented with ACC as the sole N source (**Figure 2**). In contrast, no growth was observed on N-free DF medium by this bacterium. The positive control, *Pseudomonas umsongensis* having the enzyme ACC-deaminase, showed a similar growth pattern in presence of ACC, while the negative control, *Bosea robiniae*, which lacks ACC-deaminase, failed to proliferate under ACC-supplemented conditions. None of the tested strains exhibited any growth in DF medium lacking a N source (**Figure 2A**). However, where DF medium was supplemented with ACC as the sole N source (**Figure 2B**), distinct pinkish-orange bands were visible for *M. symbioticum* SB23 and light-yellow bands for the positive control *P. umsongensis*, confirming their ability to utilize ACC for growth. The negative control, *B. robiniae*, showed no growth, verifying its inability to metabolize ACC.

### 3.4 Shoot growth and morphology

#### 3.4.1 Leaf chlorophyll content (SPAD index)

Chlorophyll content in maize leaves, quantified as SPAD units, exhibited significant temporal and spatial variations across different nitrogen (N) and *M. symbioticum* (MS) treatments from June to August 2023. Significant interaction was observed between treatments and observation time, highlighting the better impact of bacterial inoculation at the beginning and particularly at the end of the recording period.

**Figure 3:**
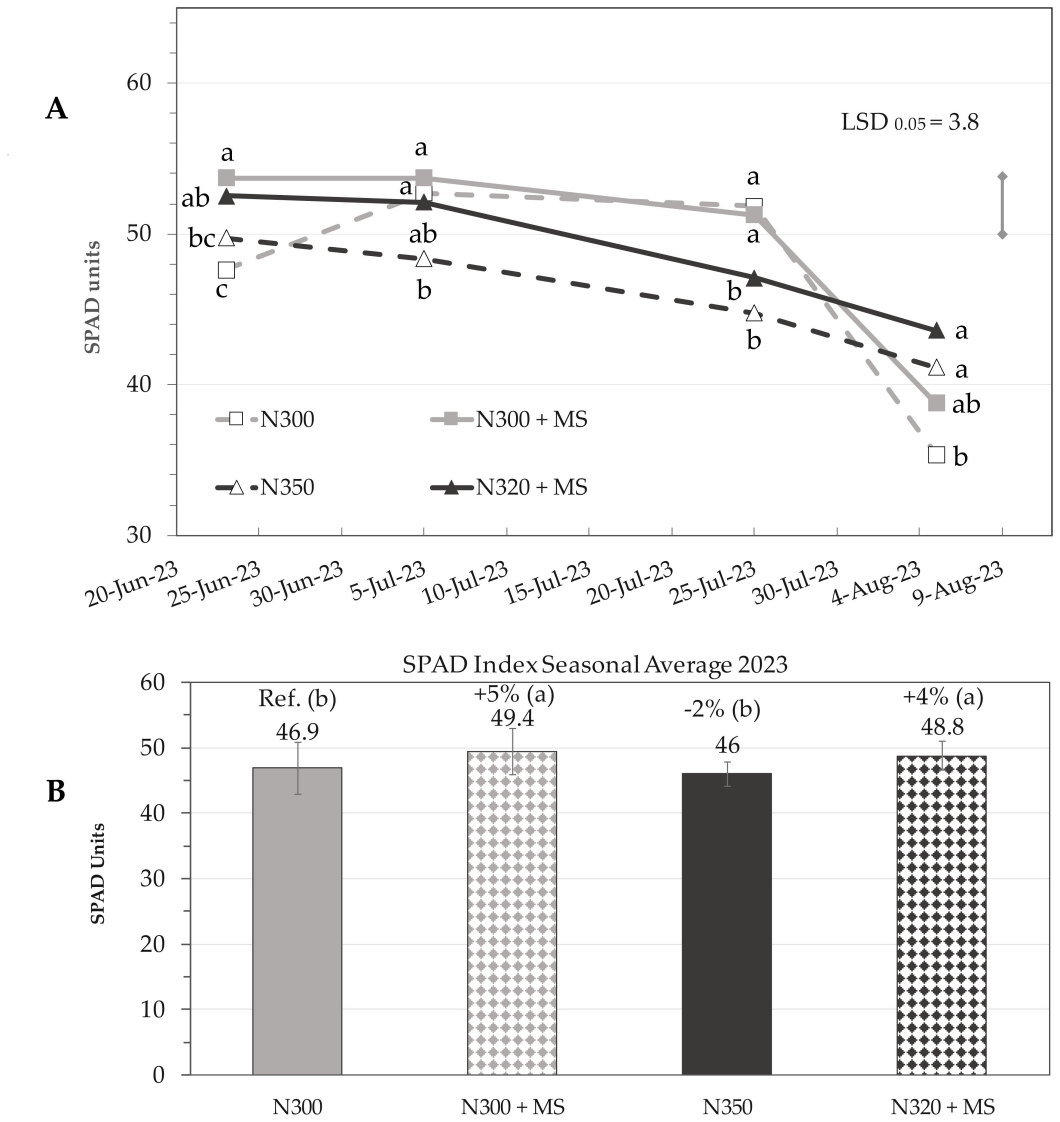
**(A)** Temporal dynamics of leaf chlorophyll content (SPAD means; *n* = 3) in maize plants subjected to different nitrogen (N) and *Methylobacterium symbioticum* (MS) treatments: N300, N300 + MS, N350 and N320 + MS. The dates of measurements in the x-axis are associated to specific phenological stages using the BBCH scale: BBCH 65 (flowering, 23 June), BBCH 73 (milking, 5 July), BBCH 85 (dough maturation, 25 July), and BBCH 87 (early physiological maturity, 5 August). The vertical bar above the graph represents the least significant difference (LSD) for the “Treatment × Time” interaction at p ≤ 0.05 (3.78). Subplot **(B)** represents the seasonal average of SPAD values (means ± SE) across the four phenological stages. The numerical value above each bar indicates the mean SPAD value for each treatment, and the percentage value denotes the relative change compared to the N300 reference. Letters in parentheses indicate statistical groupings based on Tukey’s test (p ≤ 0.05).

As an average of all leaves in a plant, a general decreasing trend of chlorophyll content was observed as the season progressed towards the harvest time (**Figure 3**). Bacterial inoculation provided a significant (p ≤ 0.05) SPAD improvement, with overall seasonal averages of N300 + MS (SPAD = 49.4) and N320 + MS (SPAD = 48.8) outperforming controls (N300; SPAD = 46.9) (**Figure 3**).

**Figure 4:**
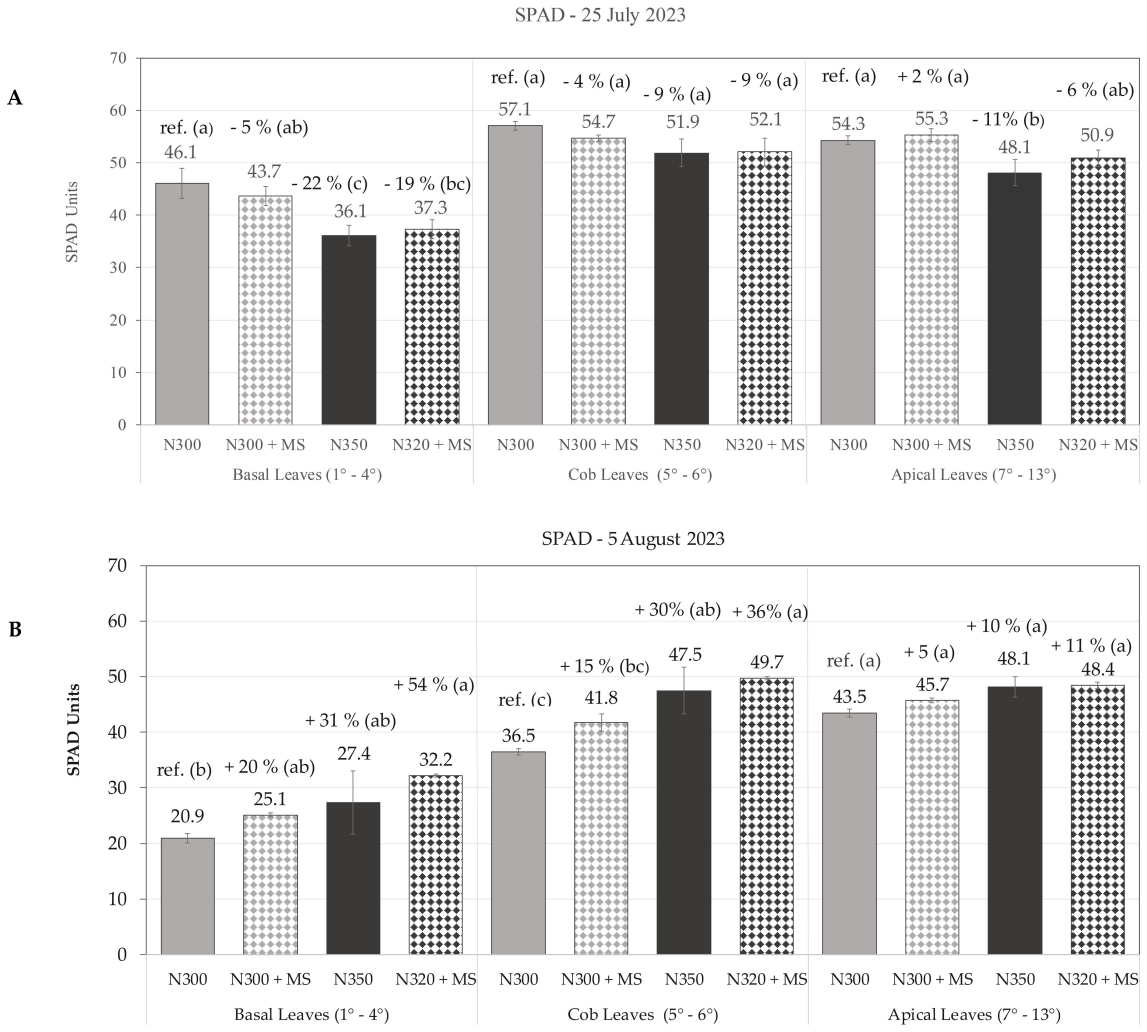
Spatial dynamics of leaf chlorophyll content, measured as SPAD units, in maize basal (1^st^-4^th^), cob (5^th^-6^th^), and apical (7^th^-13^th^) leaves at dough maturity on 25 July 2023 **(A)**, and early physiological maturity on 5 August **(B)**. The graph depicts SPAD values (mean ± se, n=3) under varying nitrogen (N) and *Methylobacterium symbioticum* (MS) treatments (N300, N300 + MS, N350, and N320 + MS) with percentage differences relative to the N300 control. Within each leaf layer, treatments with distinct letters above bars indicate significant differences (Tukey’s test, p ≤ 0.05).

By 25 July, pronounced spatial differentiation in chlorophyll content became also evident across leaf layers. In the basal (1^st^-4^th^) leaves, the control treatment (N300) exhibited the highest chlorophyll content with a mean SPAD = 46.1, significantly outperforming the N350 (−22%) and N320 + MS (−19%) treatments, while N300 + MS showed a moderate non-significant decrease of 5% (SPAD = 43.7). The N320 + MS and N350 treatments, were not significantly different from each other. Cob leaves (5^th^–6^th^) and apical leaves (7^th^–13^th^) exhibited more moderate responses, with no significant differences among treatments in cob leaves, and only slight variations in the younger apical leaves (**Figure 4**). However, the most striking observations emerged in the late-season measurements of 5 August, which revealed a pronounced stay-green effect across treatments incorporating the bacterial inoculation (MS). The N320 + MS treatment demonstrated a noteworthy response, particularly in basal leaves, where it achieved a 54% increase in SPAD values (SPAD = 32.2) compared to controls (SPAD = 20.9); cob and apical leaves of N320 + MS also showed increases of 36% (SPAD = 49.7; p ≤ 0.05) and 11% (SPAD = 48.4; n.s.), respectively, compared to controls, highlighting the ability of *Methylobacterium* to sustain chlorophyll content across all leaf layers in late-season when the plant approaches senescence. While the control treatment (N300) exhibited typical chlorophyll degradation patterns, particularly in the older basal leaves, the N300 + MS treatment showed some chlorophyll retention (SPAD = 25.1), and N320 + MS treatment was the most effective (**Figure 4**). Spatial analysis revealed a consistent gradient of treatment effectiveness across leaf layers/age, with basal and cob leaves showing the most important responses to bacterial inoculation late in the season.

#### 3.4.2 Plant morphology at the dough maturity stage

The dress application of chemical N and *M. symbioticum* significantly affected various morphological parameters of maize plants at the dough stage (19 July) (**Table 2**). The N320 + MS treatment consistently demonstrated the most pronounced improvements across multiple growth metrics, highlighting the synergistic effects of moderate N application combined with bacterial inoculation.

**Table 2:** Shoot measurements (means ± se; n=3) at the dough maturity stage (19 July) of maize across four treatments (N300, N300 + MS, N350 and N320 + MS).

Treatment	Plant height (cm)	%var/C	Number of leaves	%var/C	Leaf area index (LAI)	%var/C	Shoot biomass (g/m²)	%var/C	Number of ears per plant	%var/C
N300	235 ± 2	Ref. (b)	11.0 ± 0.3	Ref. (b)	4.1 ± 0.2	Ref. (b)	996 ± 88	Ref. (b)	1.5 ± 0.3	Ref. (a)
N300 + MS	260 ± 6	+11% (a)	12.5 ± 0.1	+14% (a)	4.5 ± 0.1	+10% (a)	1,298 ± 36	+30% (ab)	1.8 ± 0.2	+22% (a)
N350	249 ± 6	+6% (a)	11.8 ± 0.6	+8% (ab)	3.9 ± 0.1	-5% (b)	1,127 ± 93	+13% (bc)	1.8 ± 0.2	+22% (a)
N320 + MS	262 ± 3	+12% (a)	12.8 ± 0.2	+17% (a)	4.5 ± 0.1	+10% (a)	1,399 ± 39	+40% (a)	2.0 ± 0.1	+33% (a)

Letters indicate significant differences (Tukey test, p ≤ 0.05) among treatments within each parameter. Percentage variation (%var/C) shows change relative to N300 control.

Plant height increased in both inoculated treatments compared to the N300 control (p ≤ 0.05), with the N320 + MS treatment showing the greatest increase (262 cm, +12%), followed by the N300 + MS treatment (260 cm, +11%). N350 resulted in a moderate increase (249 cm, +6%), though this was less pronounced than the MS-inoculated treatments.

The number of leaves per plant also increased significantly (p ≤ 0.05) in inoculated plants, and this was reflected in the Leaf Area Index (LAI), which was significantly improved in N300 + MS and N320 + MS (both 4.5, + 10%) treatments vs. N300 control. In contrast, N350 resulted in a slight reduction in LAI (3.9, –5%).

Shoot biomass production varied accordingly, with N320 + MS reaching the highest value of 1,399 g m^–^², a 40% increase over the N300 control (996 g m^-^², p ≤ 0.05). N300 + MS also exhibited significantly greater biomass accumulation (1,298 g m^–^², +30% vs. N300) but did not differ significantly from N320 + MS or N350 (p > 0.05). Shoot biomass under N350 (1,127 g m^–^²) was not significantly different from the control (+13%) or N300 + MS (p > 0.05). This resulted in improved plant fertility, the number of ears per plant on average being slightly higher in N320 + MS, N300 + MS and N350, but not statistically different from control.

### 3.5 Root measurements

**Figure 5:**
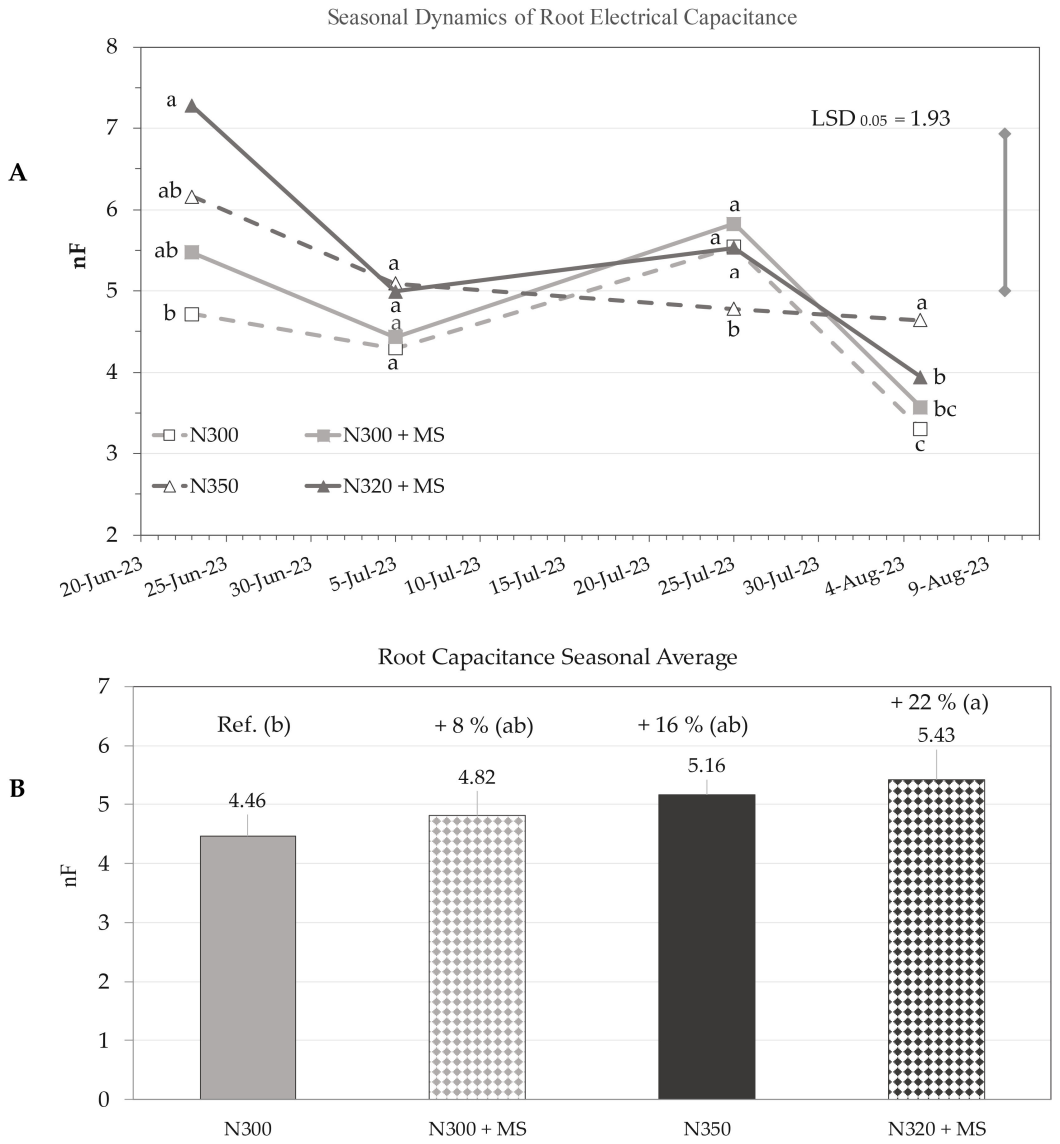
Seasonal dynamics **(A)** of root electrical capacitance (nF; mean ± se; n = 3) of maize plants under N300, N300 + MS, N350, and N320 + MS treatments at 120 Hz measurement frequency. The dates of measurements in the x-axis are associated to specific phenological stages using the BBCH scale: BBCH 65 (flowering, 23 June), BBCH 73 (milking, 5 July), BBCH 85 (dough maturation, 25 July), andBBCH 87 (early physiological maturity, 5 August). LSD bar (1.93; p ≤ 0.05) is shown for the “Treatment × Time” interaction. Subplot **(B)** illustrates the seasonal average root capacitance. Different letters indicate statistically significant differences among treatments at each stage (Tukey’s test, *p* < 0.05). Percentage values above bars in **(B)** denote changes relative to the control (N300).

Root electrical capacitance measurements revealed significant variations in root growth over time (p ≤ 0.001) across the growing season (23 June – 5 August). The ‘Treatment × Time’ interaction was not statistically significant (p > 0.05), while significant differences among treatments were observed (p ≤ 0.05). On 23 June, at maize flowering, treatments N320 + MS, N350 and N300 + MS showed +54%, +31% and +16% respectively over the control N300, although only N320 + MS was significantly higher (**Figure 5A**). At the end of the measurement period on 5 August, when approaching early physiological maturity, N350 exhibited significantly higher root electrical capacitance compared to control (N300) and N320 + MS (p ≤ 0.05). However, no statistically significant difference was observed between N350 and N320 + MS.

Across the entire measurement period (main effect ‘Treatment’), pairwise comparisons indicated that N320 + MS tended to have higher root electrical capacitance throughout the measurement period, with seasonal average significantly (p ≤ 0.05) increasing from 4.46 nF of control (N300) to 5.43 nF of N320 + MS (+22%) (**Figure 5B**). N300 + MS and N350 showed non-significant increases of 8% and 16%, respectively, compared to the control (**Figure 5**).

### 3.6 Yield and grain quality

**Figure 6:**
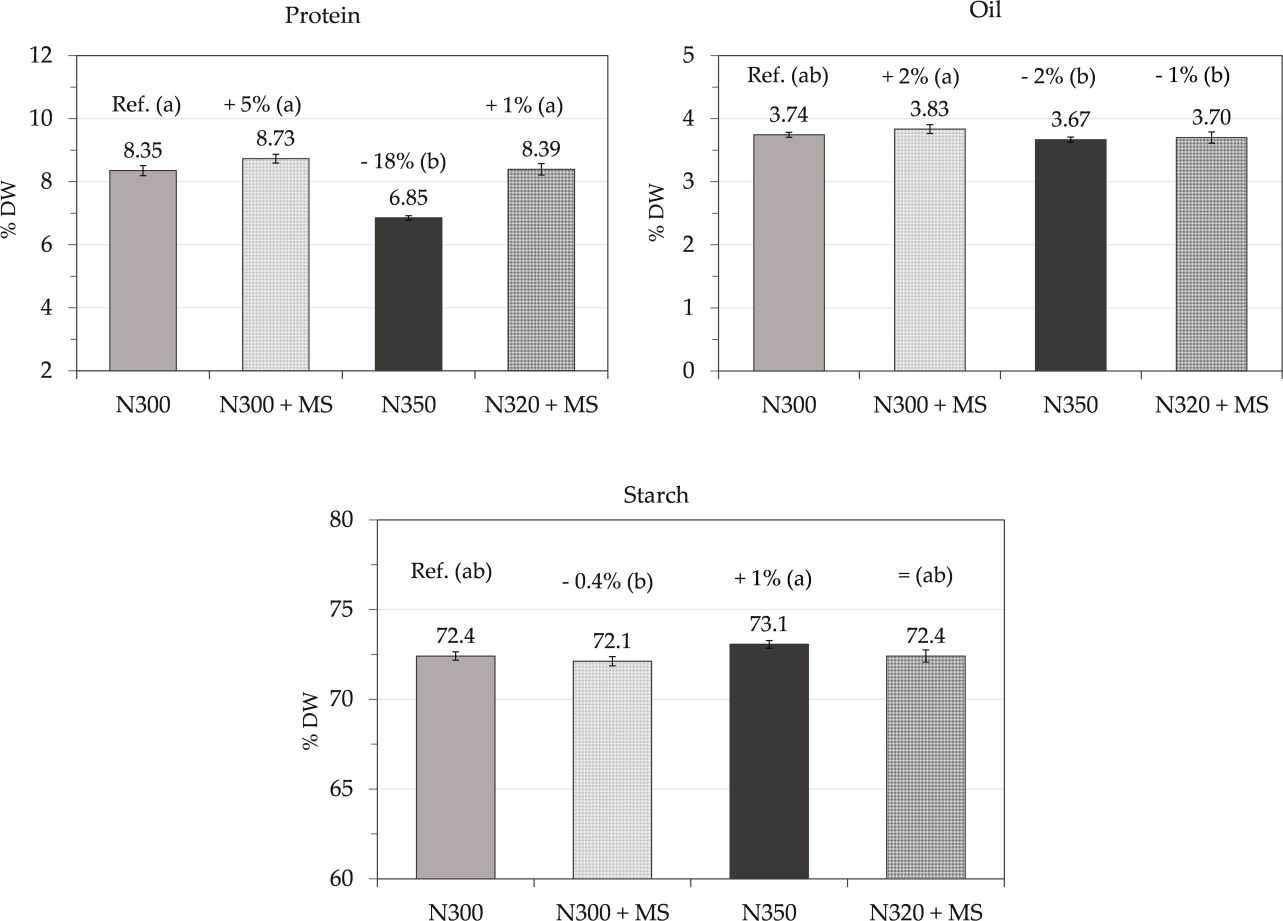
Protein, oil and starch contents (% on DW; mean ± se, n = 3) of grains at harvest measured by NIRS, in maize subjected to N300, N300 + MS, N350, and N320 + MS treatments. The values above the histograms show the means and % of variation vs. control (N300). Different letters indicate significant differences among treatments (Tukey’s test, p ≤ 0.05).

Grain yield and quality (**Table 3**; **Figure 6**) reflected some morphological differences previously observed at milk maturity. Grain yield reached the highest level under N300 + MS (1364 g m^-^²; +12% vs. N300 control at 1217 g m^-^², n.s.), and this inoculated treatment yielded significantly more than N350 (1126 g m^-^²; p ≤ 0.05). Although N320 + MS (1286 g m^-^²; +6% vs. N300) and N300 + MS both showed trended increase than the control, neither differing significantly from N300 (p > 0.05). Likewise, the 8% drop under N350 vs. N300 did not reach significance (p > 0.05).

**Table 3:** Maize yield and its components (mean ± se; n = 3) at final maturity across four treatments (N300, N300 + MS, N350, N320 + MS).

Parameter	N300	%var/C	N300 + MS	%var/C	N350	%var/C	N320 + MS	%var/C
Grain yield DW (g m^–^²)	1,217 ± 72	Ref. (ab)	1,364 ± 6	+12% (a)	1,126 ± 61	-8% (b)	1,286 ± 42	+6% (ab)
Grain moisture at harvest (%)	16.9 ± 0.4	Ref. (b)	15.6 ± 0.5	-8% (b)	19.8 ± 0.7	+17% (a)	15.9 ± 0.4	-6% (b)
TSW (g)	336 ± 56	Ref. (a)	350 ± 1	+4% (a)	339 ± 13	+1% (a)	347 ± 19	+3% (a)
Harvest Index	0.60 ± 0.01	Ref. (a)	0.58 ± 0.01	-4% (b)	0.60 ± 0.01	= (a)	0.58 ± 0.01	-4% (b)

Letters indicate significant differences among treatments (Tukey’s test, p < 0.05). Percentage variation (%var/C) shows change relative to the N300 control.

Grain moisture content at harvest varied significantly among treatments. The N300 + MS treatment showed a slight 8% reduction (15.6%) compared to the N300 control (16.9%), while N320 + MS treatment resulted in a 6% reduction (15.9%). In contrast, the N350 treatment had the highest moisture content (19.8%, +17% vs. control).

The thousand seed weight (TSW) showed slight non-significant increases in both the treatments including the bacterial inoculation N300 + MS (350 g, +4%) and N320 + MS (347 g, +3%) vs. control (336 g), while N350 resulted in a marginal increase (339 g, +1%).

The harvest index (HI) showed a slight though significant decrease in N300 + MS and N320 + MS treatments (0.58, −4%) vs. control (0.60), while N350 maintained an HI like the control (0.60).

Grain protein content showed moderate variations, with N300 + MS treatment showing a slight 5% non-significant increase (8.73% DW) relative to control (8.35% DW). However, N350 resulted in a significant 18% reduction (6.85% DW) compared to the control (p < 0.05), while N320 + MS maintained almost the same protein level (8.39%, +1%).

Oil content showed slight variations across treatments. N300 + MS resulted in a 3% increase (3.83% DW) compared to the control (3.74% DW). N320 + MS maintained oil content very similar to the control (3.70% DW), while N350 showed a slight decrease (3.66% DW, −2%). These changes, however, were not statistically significant.

Starch content remained very stable across treatments. N300 + MS showed only a slight decrease (72.1% DW, –0.4%); N350 had a minor increase (73.1%, +0.9%), while N320 + MS maintained starch levels comparable to the control (72.4%). These variations were not statistically significant.

### 3.7 Grain and crop residues elemental composition

The elemental composition of maize kernels and crop residues at harvest revealed complex responses to different N and inoculation treatments, with some significant variations observed across multiple essential elements (**Tables 4**, **5**). Grain and biomass analyses demonstrated that N management strategies, particularly the N350 and N300 + MS treatments, substantially influenced elemental accumulation patterns.

**Table 4:** Macro-elemental composition (means ± se, n = 3) of maize grains and crop residues at maturity across four treatments (N300, N300 + MS, N350, and N320 + MS).

Element	Treatment	Grains (mean ± se)	% var/C	Crop residues (mean ± se)	% var/C
N (%)	N300 N300 + MS N350 N320 + MS	1.34 ± 0.03 1.40 ± 0.02 1.10 ± 0.01 1.34 ± 0.03	Ref. (a) + 5% (a) – 18% (b) + 1% (a)	0.91 ± 0.05 1.05 ± 0.08 0.69 ± 0.05 0.89 ± 0.12	Ref. (ab) + 15% (a) – 24% (b) – 3% (ab)
Ca (ppm)	N300 N300 + MS N350 N320 + MS	39.9 ± 0.7 40.9 ± 3.3 46.1 ± 2.3 40.6 ± 2.9	Ref. (a) + 3% (a) + 15% (a) + 2% (a)	6,196 ± 575 7,145 ± 640 5,014 ± 175 6,116 ± 1,357	Ref. (a) + 15% (a) – 19% (a) – 1% (a)
K (ppm)	N300 N300 + MS N350 N320 + MS	3,212 ± 111 3,203 ± 31 3,376 ± 13 3,164 ± 69	Ref. (a) – 0.3% (a) + 5% (a) – 2% (a)	11,454 ± 254 12,212 ± 429 13,384 ± 392 13,016 ± 446	Ref. (b) + 7% (ab) + 17% (a) + 14% (a)
Mg (ppm)	N300 N300 + MS N350 N320 + MS	958 ± 9 991 ± 14 889 ± 19 921 ± 12	Ref. (ab) + 4% (a) – 7% (c) – 4% (bc)	3,057 ± 159 2,616 ± 253 2,151 ± 137 2,206 ± 272	Ref. (a) – 15% (ab) – 30% (b) – 28% (b)
P (ppm)	N300 N300 + MS N350 N320 + MS	2,755 ± 69 2,713 ± 32 2,812 ± 53 2,546 ± 41	Ref. (a) – 2% (a) + 2% (a) – 8% (b)	1,462 ± 106 1,429 ± 128 1,871 ± 140 1,118 ± 99	Ref. (b) – 2% (b) + 28% (a) – 24% (b)
S (ppm)	N300 N300 + MS N350 N320 + MS	1,019 ± 18 1,099 ± 20 988 ± 3 1,055 ± 15	Ref. (bc) + 8% (a) – 3% (c) + 4% (ab)	1,297 ± 100 1,543 ± 127 1,467 ± 25 1,361 ± 202	Ref. (a) + 19% (a) + 13% (a) + 5% (a)

Nitrogen is expressed as % dry weight; all other elements as mg kg^-^¹ (ppm). Within each element, different letters indicate statistically significant differences among treatments, and percentage changes (% var/C) variations relative to the N300 control (C) (Tukey’s test, p ≤ 0.05).

**Table 5:** Micro-elemental composition (means ± se, n = 3) as mg kg^-^¹ (ppm) of maize grains and crop residues at maturity across four treatments (N300, N300 + MS, N350, and N320 + MS).

Element	Treatment	Grains (mean ± SE)	% var/C	Crop residues (mean ± SE)	% var/C
Cu (ppm)	N300 N300 + MS N350 N320 + MS	1.51 ± 0.07 1.44 ± 0.01 1.68 ± 0.05 1.50 ± 0.11	Ref. (ab) – 5% (b) + 1%1 (a) – 1% (ab)	11.0 ± 1.5 13.3 ± 1.9 7.8 ± 0.5 12.0 ± 2.5	Ref. (a) + 21% (a) – 29% (a) + 9% (a)
Fe (ppm)	N300 N300 + MS N350 N320 + MS	16.3 ± 0.4 19.7 ± 2.1 18.2 ± 1.4 16.6 ± 0.3	Ref. (a) + 21% (a) + 12% (a) + 2% (a)	68.5 ± 12.7 114.0 ± 15.0 83.6 ± 6.6 106.0 ± 16.9	Ref. (b) + 67% (a) + 22% (ab) + 55% (ab)
Mn (ppm)	N300 N300 + MS N350 N320 + MS	2.33 ± 0.06 2.31 ± 0.12 3.34 ± 0.03 2.78 ± 0.25	Ref. (bc) – 1% (c) + 43% (a) + 19% (b)	4.77 ± 0.61 5.93 ± 0.58 7.69 ± 0.34 6.00 ± 0.89	Ref. (b) + 24% (ab) + 61% (a) + 26% (ab)
Na (ppm)	N300 N300 + MS N350 N320 + MS	27.8 ± 1.6 22.2 ± 3.5 20.1 ± 4.1 20.0 ± 2.9	Ref. (a) – 20% (a) – 28% (a) – 28% (a)	50.6 ± 5.8 51.9 ± 3.2 49.4 ± 3.0 54.8 ± 8.2	Ref. (a) + 3% (a) – 2% (a) + 8% (a)
Zn (ppm)	N300 N300 + MS N350 N320 + MS	19.5 ± 0.1 19.7 ± 0.4 21.7 ± 0.4 20.0 ± 0.3	Ref. (b) + 1% (b) + 11% (a) + 3% (b)	30.0 ± 3.1 34.0 ± 4.1 43.3 ± 1.6 32.6 ± 2.7	Ref. (b) + 14% (ab) + 44% (a) + 9% (b)

Within each element, different letters indicate statistically significant differences among treatments, and percentage changes (% var/C) variations relative to the N300 control (C) (Tukey’s test, p ≤ 0.05).

Regarding N concentration, aligned with grain protein content, a significant unexpected decrease was observed in treatment N350. Conversely, N300 + MS exhibited the most notable (n.s.) increment (+5% vs. N300 control). Kernel Ca content showed a notable increase in the N350 treatment (+15% vs. control; n.s.), while Cu exhibited similar trends with +11% in the same treatment, which was statistically significant vs. control.

Fe content was particularly responsive, with the N300 + MS having a +21% vs. control, but again it was only a trend. The most significant change was observed in Mn, where N350 induced a remarkable +43% in grain concentration. K and P remained relatively stable, with only minor fluctuations, although –8% was observed in N320 + MS for grain P.

Regarding the elemental composition of crop residues, there were more pronounced treatment effects compared to grains. N content was notably lower in N350 (-24%) compared to N300 control, aligning with trends observed in the grains, meanwhile, N300 + MS showed a moderate non-significant increase (+15%) and N320 + MS a minor decrease (−3%).

Ca levels increased non-significantly by 15% in N300 + MS. Fe content showed substantial alterations, with N300 + MS and N320 + MS increasing by 67% (p ≤ 0.05) and 55% vs. N300, respectively. K exhibited significant increases, with N350 and N320 + MS treatments showing +17% and +14% vs. control, respectively.

Mn demonstrated notable responsiveness, with N350 increasing biomass Mn by 61%. Conversely, some treatments resulted in decreased levels of certain elements, particularly Mg and P. The N350 treatment, for instance, induced a –30% in Mg (p ≤ 0.05), while N320 + MS showed a −24% non-significant decrease in P content.

The application of *Methylobacterium symbioticum* significantly enhanced the overall N accumulation across grains and biomass of maize under varying N levels (**Supplementary Table S1**). N300 + MS showed the greatest improvement, with grain N increasing by 17%, crop residues by 51%, and overall N accumulation by 30% vs. N300 control. The N320 + MS treatment also improved N uptake (+6% grains, +37% crop residues, +17% total), though less effectively than N300 + MS. Conversely, N350 led to a decrease in N accumulation (-24% grains, -14% crop resides, -20% total). Statistical analysis confirmed that N300 + MS was the most effective treatment, followed by N320 + MS, while N350 had the lowest N efficiency.

### 3.8 Correlation analysis

Correlation analysis revealed significant relationships between various plant parameters (**Table 6**). Plant height exhibited strong positive correlations with vegetative growth metrics, including leaf count (r = +0.88***, p ≤ 0.001) and LAI (r = +0.66*, p ≤ 0.05). Furthermore, plant height strongly correlated with total shoot biomass (r = +0.86***, p ≤ 0.001) and moderately with grain yield (r = +0.50, n.s.). LAI emerged as a critical factor influencing grain yield (r = +0.82**, p ≤ 0.01) and demonstrated a significant correlation with ear count (r = +0.58*, p ≤ 0.05). Seasonal SPAD values showed a significant negative correlation with the harvest index (r = –0.78**, p ≤ 0.01). Root electrical capacitance exhibited positive weak correlations with plant height (r = +0.21, n.s.) and LAI (r = +0.13, n.s.), the positive trend being consistent with our field observations of enhanced root development in *Methylobacterium*-treated plants, particularly in N320 + MS. Among yield components, grain yield positively correlated with TSW (r = +0.63*, p ≤ 0.05) and negatively with grain moisture (r = –0.52, n.s.). Among grain quality parameters, grain protein content showed a negative correlation with oil content (r = –0.28, n.s.).

**Table 6:** Pearson correlation coefficients (r) between morphological, physiological, and yield parameters in maize across four treatments (N300, N300 + MS, N350, and N320 + MS).

	SPAD	Root capacitance	Plant height	No. of leaves	LAI	Shoot biomass	No. of ears	Grain yield	Grain moisture	TSW	HI	Protein %	Oil %	Starch %
SPAD		+0.11 ns	+0.24 ns	+0.05 ns	+0.36 ns	+0.24 ns	+0.04 ns	+0.37 ns	–0.52 ns	+0.09 ns	–0.78**	–0.32 ns	+0.37 ns	–0.40 ns
Root capacitance	+0.11 ns		+0.21 ns	+0.30 ns	+0.13 ns	+0.17 ns	+0.22 ns	–0.18 ns	–0.05 ns	–0.08 ns	–0.37 ns	+0.30 ns	+0.04 ns	+0.49 ns
Plant height	+0.24 ns	+0.21 ns		+0.88***	+0.66*	+0.86***	+0.74**	+0.50 ns	–0.27 ns	+0.32 ns	–0.52 ns	–0.28 ns	+0.02 ns	–0.24 ns
No. of leaves	+0.05 ns	+0.30 ns	+0.88***		+0.70*	+0.88***	+0.64*	+0.48 ns	–0.34 ns	+0.41 ns	–0.53 ns	–0.14 ns	+0.00 ns	–0.26 ns
LAI	+0.36 ns	+0.13 ns	+0.66*	+0.70*		+0.57 ns	+0.58*	+0.82**	–0.74**	+0.34 ns	–0.54 ns	–0.23 ns	+0.41 ns	–0.64*
Shoot biomass	+0.24 ns	+0.17 ns	+0.86***	+0.88***	+0.57 ns		+0.45 ns	+0.39 ns	–0.42 ns	+0.33 ns	–0.61*	–0.19 ns	–0.06 ns	–0.27 ns
No. of ears	+0.04 ns	+0.22 ns	+0.74**	+0.64*	+0.58*	+0.45 ns		+0.40 ns	–0.04 ns	+0.38 ns	–0.17 ns	+0.05 ns	–0.14 ns	–0.18 ns
Grain yield	+0.37 ns	–0.18 ns	+0.50 ns	+0.48 ns	+0.82**	+0.39 ns	+0.40 ns		–0.52 ns	+0.63*	–0.52 ns	–0.45 ns	+0.39 ns	–0.86***
Grain moisture	–0.52 ns	–0.05 ns	–0.27 ns	–0.34 ns	–0.74**	–0.42 ns	–0.04 ns	–0.52 ns		–0.03 ns	+0.52 ns	+0.34 ns	–0.57 ns	+0.54 ns
TSW	+0.09 ns	–0.08 ns	+0.32 ns	+0.41 ns	+0.34 ns	+0.33 ns	+0.38 ns	+0.63*	–0.03 ns		–0.37 ns	–0.12 ns	–0.28 ns	–0.59*
HI	–0.78**	–0.37 ns	–0.52 ns	–0.53 ns	–0.54 ns	–0.61*	–0.17 ns	–0.52 ns	+0.52 ns	–0.37 ns		+0.28 ns	–0.31 ns	+0.41 ns
Protein %	–0.32 ns	+0.30 ns	–0.28 ns	–0.14 ns	–0.23 ns	–0.19 ns	+0.05 ns	–0.45 ns	+0.34 ns	–0.12 ns	+0.28 ns		–0.65*	+0.36 ns
Oil %	+0.37 ns	+0.04 ns	+0.02 ns	+0.00 ns	+0.41 ns	–0.06 ns	–0.14 ns	+0.39 ns	–0.57 ns	–0.28 ns	–0.31 ns	–0.65*		–0.24 ns
Starch %	–0.40 ns	+0.49 ns	–0.24 ns	–0.26 ns	–0.64*	–0.27 ns	–0.18 ns	–0.86***	+0.54 ns	–0.59*	+0.41 ns	+0.36 ns	–0.24 ns	

Significance levels: *p ≤ 0.05; **p ≤ 0.01; ***p ≤ 0.001; ns, not significant.

## 4 Discussion

### 4.1 Maize-*Methylobacterium symbioticum* interaction

The application of the nitrogen-fixing bacterium *Methylobacterium symbioticum* presents a promising advancement in sustainable cultivation of non-legume crops like maize. The present study investigates the effects of foliar applied *M. symbioticum* under different N fertilization regimes on key performance parameters of maize, such as vegetative growth, root growth, yield, and nutrient content. This trial confirmed that inoculation with *M. symbioticum* can enhance maize growth and the quality of grain by partially reducing the dependence on chemical fertilizers, as the best improvements were achieved when combining moderate N rates with bacterial inoculation These findings are consistent with recent studies on wheat and maize, where *M. symbioticum* application under reduced N regimes maintained or improved growth and yield, suggesting a consistent benefit of this bacterium across cereal crops under suboptimal N supply. However, some field studies have reported limited effects of *M. symbioticum* on cereal yield, highlighting that environmental conditions and management practices can affect the magnitude of the response (Torres Vera et al., 2024; Valente et al., 2024).

The development of effective nitrogen-fixing systems is a critical challenge in sustainable agriculture. Unlike traditional symbiotic N fixation of legumes, cereal crops like maize lack the specialized structures necessary for these associations (Pankievicz et al., 2019; Guo et al., 2023). Consequently, there is an increasing interest in plant-associated bacteria that can aid plants by fixing N and promoting growth. It has been demonstrated that *M. symbioticum* establishes beneficial plant associations without the requirement for specialized root nodules (Zhang et al., 2021). In this way, the microbial approach could partially address some environmental concerns linked to chemical N fertilizers, such as greenhouse gas emissions, nitrate water pollution, and soil degradation (Galloway et al., 2008). This is similar to the benefits reported for other plant growth-promoting bacteria (PGPB) such as *Azospirillum* and *Bacillus* spp., which have been widely studied in maize and wheat for their ability to improve nitrogen use efficiency and reduce fertilizer supply (Barbosa et al., 2022).

The performance and success of nitrogen-fixing inoculants depends on environmental conditions, also including the dose of N fertilization. Research indicates that such inoculants improve nitrogen use efficiency and plant growth through direct N fixation, improved nutrient scavenging, and hormonal modulation (Gómez-Duque et al., 2022; Timofeeva et al., 2023). These processes are compatible with current knowledge on various plant growth-promoting bacteria (PGPB), which contribute to crop productivity via both direct metabolic activities and indirect effects on plant physiology and tolerance to stress (Gamalero and Glick, 2019). By combining foliar application of *M. symbioticum* under varying N regimes, it was possible to assess its potential to substitute or supplement synthetic N inputs while maintaining crop productivity (Berendsen et al., 2012). This integrated strategy highlights that microbial interventions must be tailored to complement conventional fertilization at a suitable rate, especially under high-yielding systems where exclusive reliance on biological N fixation might not be feasible in the short term.

This study, by employing ESEM under aseptic conditions, revealed efficient colonization of maize leaf surface by this *M. symbioticum* strain. The bacterium primarily aggregates along foliar veins and near stomatal openings, indicating possible easy penetration into leaf tissue of this crop species while remaining extracellularly and maintaining proximity to these key structural sites (Zhang et al., 2021).

### 4.2 Vegetative growth responses

At the dough stage, maize inoculated with *M. symbioticum* exhibited significantly enhanced vegetative growth, especially when combined with a moderate level of N fertilization, outperforming control plants that received a standard N rate. Although increases in plant height across treatments were modest with no statistical significance, other crucial vegetative parameters demonstrated pronounced improvements with bacterial inoculation. Importantly, LAI increased when plants received the bacterial treatment regardless of the dose of chemical N fertilization. This provides plants with greater radiation use and photosynthetic capacity during critical stages of development, and may be attributed to bacterial phytohormone production, specifically cytokinins and auxins, which have been recognized to stimulate cell division and primordia development in leaves of cereal crops (Palberg et al., 2022; Wu et al., 2021). Plants treated with the bacterial inoculant, in combination with either conventional (N300) or moderate (N320) N fertilization, achieved LAI values of 4.5, reflecting significant improvements of 10% over controls, which is a key determinant of biomass accumulation. The magnitude of LAI improvement is especially relevant, considering that even small increases in light interception during critical periods of maize growth can have a great influence on the final yield of this C4 species (Rodrigues et al., 2024; Torres Vera et al., 2024). LAI increases are similar to those observed in maize treated with *Azospirillum brasilense* under moderate N fertilization, as reported by Barbosa et al. (2022), further indicating that microbial inoculation can enhance canopy development in maize under specific conditions.

Total shoot biomass also exhibited marked enhancements in response to microbial inoculation. At the dough stage of maturation, the N320 + MS treatment peaked at 1,399 g DW m^–^² (40% increase over N300 controls), while N300 + MS achieved 1,298 g m^–^² (30% increase). This suggests that *M. symbioticum* can optimize N assimilation and resource allocation even in later stages from its application, potentially by enhancing N accumulation, improving metabolic efficiency, or modulating hormonal balance to favor shoot development, together with photosynthetic efficiency and carbon allocation, like observed in wheat by Valente et al. (2024) with the same bacterial strain.

Notably, both the treatments including the bacterial inoculation enhanced ear differentiation and reproductive structure development, which had direct implication on yield. This may be the result of enhanced assimilation during the critical ear differentiation period at the beginning of stem elongation (stage V8-V10; i.e, 8-10-leaf stage) (Torres Vera et al., 2024; Liu et al., 2022). Similar improvements in shoot biomass and reproductive development have been observed in wheat and rice following *M. symbioticum* application, although the magnitude of yield response in maize appears more variable, likely due to differences in crop physiology and environmental interactions (Zhang et al., 2021).

### 4.3 Chlorophyll status and SPAD dynamics

The analysis of the overall plant chlorophyll content using SPAD measurements revealed complex temporal and spatial dynamics. Early-season observations showed inferior photosynthetic capacity at the lowest N fertilization without bacterial inoculation, while variable benefits were constantly recorded by the application of *M. symbioticum* throughout the measurement period, as highlighted by the higher seasonal SPAD averages. The effects of bacterial inoculation on leaf chlorophyll content became even more evident when approaching maturity and plant senescence, focusing on basal leaves (positions 1-4) on 25 July, and cob leaves and apical leaves later. This suggests that this bacterium exerts a stay-green effect that prolongs photosynthetic activity during critical grain development stages. The observed differential response among leaf positions suggests that *M. symbioticum* influences the natural senescence process, which starts from older basal leaves through localized effects on N metabolism enzymes and senescence-associated gene expression (Grossi et al., 2024). Such persistent photosynthetic activity has been frequently demonstrated in studies investigating bio-stimulant effects on crop physiology, regardless of the crop species (Rouphael and Colla, 2020). The N320 + MS treatment consistently maintained the maximum chlorophyll concentration in the basal leaves, while also showing considerable increases of 36% in cob leaves and 11% in apical leaves compared to controls. This is likely because of the combined effect of delayed senescence from both N fertilization and bacterial application.

### 4.4 Mechanistic insights into ACC-deaminase and hormonal modulation

The ACC deaminase activity observed in *M. symbioticum* SB23 provides a plausible mechanistic basis for the delayed leaf senescence and prolonged chlorophyll retention in maize. By breaking down ACC into α-ketobutyrate and ammonia, the enzyme lowers ethylene biosynthesis, which is a crucial phytohormone for triggering senescence—thus maintaining chlorophyll stability in older leaves (Shahid et al., 2023). This ethylene attenuation appears to operate synergistically with enhanced nitrogen metabolism, as reduced ethylene levels are known to upregulate key N-assimilating enzymes such as glutamine synthetase and glutamate synthase, facilitating improved amino-N assimilation and remobilization during grain filling (Grossi et al., 2024). In addition to modulating ethylene and N, ACC deaminase-mediated ethylene reduction has been found to strengthen the plants antioxidant machinery. PGPR-inoculated maize and other cereals generally have greater superoxide dismutase, catalase, and peroxidase activities, resulting in decreased ROS buildup, and reduced lipid peroxidation with greater membrane integrity during drought and salt stress (Al-Turki et al., 2023; Shabaan et al., 2022).

Similar findings were reported in wheat inoculated with the same bacterial strain, where ACC deaminase activity was associated with extended photosynthetic activity and improved N metabolism under reduced fertilization regimes (Valente et al., 2024). However, despite the delayed senescence, yield improvements in maize were only marginal, and this may reflect inherent limitations in the source-sink dynamics and warrants further investigation into crop-specific ethylene regulation.

Recent transcriptomic analyses have revealed leaf-position–specific regulation of senescence-associated gene expression and chlorophyll catabolism pathways (Zhen et al., 2024), offering a conceptual framework that may be applicable to PGPR-mediated stay-green effects in maize. Several PGPR strains, including *Methylobacterium*, are also found to increase cytokinin production or transport, stabilizing chloroplast membranes and photosystem II activity during grain filling. This cytokinin-mediated impact has been reported in a variety of *Methylobacterium* species, which produce significant quantities of trans-zeatin and other active CK forms that affect photosynthetic machinery and stress resistance (Palberg et al., 2022).

Enhanced chlorophyll retention throughout the canopy suggests that *M. symbioticum* may also elevate some phytohormones, such as jasmonic and salicylic acid, that are reported to delay senescence processes and retain photosynthetic capacity during reproductive stages, thus enhancing stress resilience and improve N remobilization efficiency during grain filling, as proposed by recent models (Chen et al., 2024; Yim et al., 2014). Collectively, these findings suggest that ACC deaminase activity, coupled with phytohormonal modulation and enhanced N metabolism, may extend the photosynthetically active period and contribute to yield stability under variable climatic conditions (Singh et al., 2022).

### 4.5 Root development

Non-destructive root electrical capacitance (EC) readings revealed insights into below-ground responses of maize to N fertilization and *M. symbioticum* treatments as supported by the seasonal trends in the absence of ‘Treatment × Time’ interaction.

Across both the initial (23 June) and final (5 August) measurements dates, plants treated with N320 + MS treatment displayed consistently higher root EC values, with a seasonal average 22% greater than the control. This indicates an extended additional positive effect of *M. symbioticum* inoculation, especially at 320 kg N ha^-^¹, which may be associated with ACC deaminase’s role in stress mitigation, as reduced ethylene levels can also promote root growth under suboptimal N conditions (Torres Vera et al., 2024; Valente et al., 2024; Cseresnyés et al., 2018; Rousk et al., 2010).

Mechanistically, *Methylobacterium symbioticum* could improve root functional capacity under high nitrogen levels via several, coordinated processes. It can produce indole-3-acetic acid (IAA), which stimulates lateral root formation and increases the root-soil contact (Patten and Glick, 2002). Furthermore, it secretes exopolysaccharides that contribute to rhizosphere aggregation and modify soil physicochemical properties, including dielectric characteristics, thereby improving water retention and ion exchange (Pham et al., 2024) which further optimize EC readings.

Peak EC values in N320 + MS were reported in late July, probably due to cumulative phytohormonal effects that promote long-term root activity (Grossi et al., 2024). The subsequent EC decline noticed after July is most likely due to decreasing soil moisture during late-season drying, since EC measurements are sensitive to root-soil contact and soil moisture (Cseresnyés et al., 2018; Dietrich et al., 2013). Despite this constraint, the EC advantage of N320 + MS is consistent with previous research associating *Methylobacterium* spp. to root growth promotion through phytohormonal regulation (Torres Vera et al., 2024). However, studies specifically on assessing the effect of the *M. symbioticum* on root growth effects remain limited, while other PGPR, such as *Azospirillum*, have been shown to improve root expansion through the production and exchange of auxins through root associations in many cereal crops. However, very recently Valente et al. (2024) have demonstrated that this *Methylobacterium* strain can also increase root length density in the arable layer in common wheat in open field.

Excessive chemical N fertilization generally limits root growth in cereal plants (Chen et al., 2020; Yokamo et al., 2023; Ren et al., 2024). As the maximum effects on root electrical capacitance of *Methylobacterium* was observed at high N doses, such response seems positive to counteract the negative impact of N fertilization on root growth thereby increasing the plant’s tolerance to stress conditions.

While EC is correlated with root biomass, the data must be interpreted with caution because environmental factors, particularly soil moisture, have a significant influence on EC measurements (Cseresnyés et al., 2018; Ellis et al., 2013; Dong et al., 2023). In the early stages of the trial, EC values were elevated, likely due to the maximum root expansion into moist soil occurring usually at flowering in maize. Then, over time, a decreasing EC trend was observed which may seem inconsistent with actual root growth but could be attributed to root aging and the declining soil moisture during summer, which limits root-soil water-electrode contact.

Enhanced root EC was associated with improved above-ground performance, as N320 + MS treated plants also had higher shoot height, biomass and LAI. This indicates that a more developed root system is directly linked to an increased aerial growth and yield by facilitating resource acquisition and translocation. This suggests that *M. symbioticum* might cause whole-plant physiological changes that optimize resource allocation and use throughout the growth season (Kou et al., 2022; Kitchen et al., 2003).

### 4.6 Grain yield and quality parameters

As a result of the beneficial effects on vegetative and root growth, *M. symbioticum* affected final grain yield and quality. The inoculated N300 + MS treatment increased grain yield by 12% compared to N300 control; however, this difference was not statistically significant, indicating a positive trend that warrants additional investigation, and thereby also indicating that bacterial inoculation could potentially boost productivity even without additional N inputs. As the effect of N320 + MS was less evident in terms of yield (+6%), it is suspected that crop performance may not follow a simple linear relationship with N availability when bacterial inoculants are applied. The N350 treatment displayed a statistically significant 8% reduction in yield relative to N300 + MS (p ≤ 0.05), while its difference with control was not significant, suggesting that excessive N application may negatively impact productivity when not balanced with biological input (Galindo et al., 2024). Ultimately, this bacterium may promote grain yield in maize at lower doses of N supplied, in agreement with other studies on maize, wheat, rice, grapevine, and strawberry (Pascual et al., 2020; Valente et al., 2024). However, it is important to note that while some studies report significant yield increases with *M. symbioticum* or other PGPB in maize, others find more moderate or variable effects, likely due to differences in genotype, environment, and agronomic management (Rodrigues et al., 2024; Torres Vera et al., 2024).

Grain quality parameters showed similarly promising trend responses to bacterial inoculation. Although associated with retarded leaf senescence, bacterial inoculation positively provided lower grain moisture content at harvest than controls, suggesting efficient resource remobilization during grain filling and faster dry down (Malik and Rengel, 2013), that potentially allows for improved post-harvest management and reduced drying costs. Additionally, grain protein/N content increased by 5% in the N300 + MS treatment, thus enhancing, although as a trend, the nutritional value of the harvested product. The bacteria might promote more efficient N metabolism by increasing the activity of enzymes like glutamine synthetase and glutamate synthase (Fortunato et al., 2023). However, major increases in N content were observed in crop residues of the N300 + MS treatment, a result that might also be related to the N-fixation ability of this bacterium (Madhaiyan et al., 2015), although we did not investigate this aspect in the current study. In contrast, the N350 treatment showed a 18% decrease in grain protein/N content and even more in the crop residues (−24%), despite the higher N supply, emphasizing the potential negative effects of excessive N application in the absence of microbial assistance (Galindo et al., 2024). Minor fluctuations in oil, starch, and TSW, indicate that biomass partitioning remained balanced in bacterial-treated plants.

Elemental composition analysis revealed subtle shifts in nutrient uptake and a minor role of the bacterium. While *M. symbioticum* moderately increased Fe content in grains (+21% in N300 + MS)–a favorable effect in Fe-poor grains of many cereals including maize–and K in crop residues (+14% in N320 + MS), it decreased Mg and P levels particularly in crop residues. These contrasting responses suggest that bacterial inoculation may alter nutrient competition or translocation pathways. For example, improved rhizosphere solubilization might boost Fe and Mn uptake, whereas decreased Mg could indicate cation balance trade-offs or antagonism with K. This highlights the complex relationship between microbial-mediated nutrient mobilization and root architectural modifications, or interactions with nutrient translocation (Rodrigues et al., 2024; Singh et al., 2020). Such nutrient shifts have also been observed in other studies on PGPB in cereals, suggesting that microbial inoculation can affect not only N dynamics but also the uptake and partitioning of other essential elements (Timofeeva et al., 2023; Chen et al., 2024). Variations in elemental composition highlight the multifaceted nature of microbial influence on nutrient uptake and partitioning. While some changes were statistically significant, others showed moderate trends; therefore, results must be interpreted with care to avoid overestimating bacterial impact.

Importantly, the foliar application of *M. symbioticum* significantly increased N accumulation in maize grain and biomass, particularly at the lowest N dose (N300 + MS treatment), surpassing even the higher mineral N input (N350). These findings would further sustain the role of this bacterium in plant N metabolism and use efficiency, likely through a combination of various effects, such as biological N fixation and improved N assimilation, and enhanced root growth and function, as supported by the morpho-physiological data.

The correlation analysis confirms the important role of *M. symbioticum* inoculation, as LAI enhancement caused by this bacterium is strongly correlated with grain yield (r = +0.82; p ≤ 0.05), while the stay-green effect (as seasonal SPAD) (r = + 0.35, n.s.) and root capacitance enhancement (r = –0.18, n.s.) were poorly correlated with productivity. Instead, the negative correlation between seasonal SPAD and harvest index (r = –0.78, p ≤ 0.01) suggests to further investigate the source-sink competition related to the stay-green effect under *M. symbioticum* application.

### 4.7 Environmental and economic implications, limitations and future perspectives

From an environmental standpoint, integrating *Methylobacterium symbioticum* into maize production systems offers numerous benefits. This biological approach can potentially allow a reduction in environmental contamination due to excess N fertilization. Conventional N fertilization frequently results in nitrate leaching and groundwater pollution, nitrous oxide emissions in the atmosphere which contribute to climate change, and ammonia volatilization which impairs air quality (Galloway et al., 2008; Robertson and Vitousek, 2009). The moderate yield improvements found in MS-treated plots, which were obtained with lower N inputs, imply that bacterial inoculation can minimize these environmental issues while maintaining or even increasing agricultural production.

Moreover, this study is one of the first to demonstrate the effective foliar application of *Methylobacterium symbioticum* as a biofertilizer in open-field maize agriculture. Unlike conventional soil or seed inoculation procedures, foliar administration allowed for direct colonization of the maize phyllosphere, as evidenced by environmental scanning electron microscopy (ESEM). This strategy increased physiological metrics like chlorophyll retention and root activity while also providing a unique avenue for targeted nitrogen-fixing bacteria administration under changeable field circumstances. Foliar inoculation, which bypasses soil microbial interactions, may improve delivery efficiency and consistency, making it a viable option for advancing sustainable nitrogen management practices.

Economically, the use of *M. symbioticum* could potentially reduce fertilizer requirements, which is a significant benefit given the rising and variable costs of N fertilizers. For example, the N300 + MS treatment boosted yield by 12% while using the same N application as the N300 control, thus increasing the return on fertilizer investment. Similarly, the N320 + MS treatment produced yields comparable to higher N inputs (N350), demonstrating that farmers could lower fertilizer expenses while maintaining productivity. Other potential advantages may include improved grain quality, possibly leading to higher market prices, and lower grain drying costs due to lower moisture content at harvest. On the other hand, the application costs of these bacterial inoculants can be minimized when applied along with the post-emergence weeding, in this way saving time and fuel costs. Although any possible interaction between the bacterium and herbicides requires preliminary investigation, at this time the cost-effectiveness would lie in the compensation point between the cost of the applied biofertilizer with that of saved chemical fertilizer, at least in conventional (but not in organic) management systems.

While the current study highlights the potential of foliar-applied *Methylobacterium symbioticum* to maintain maize productivity under reduced nitrogen inputs, its wider relevance would benefit from multi-location, multi-season trials with larger plot scales and increased replication to verify treatment stability and statistical robustness across diverse environments. Concurrently, field-level investigations of bacterial colonization dynamics and formulation persistence are required to assure long-term effectiveness in commercial production settings.

Further research should clarify the biological nitrogen fixation and nutrient intake processes that cause observed mineral composition changes, therefore maximizing yield-quality trade-offs.

Future studies should also include direct measurement and quantification of biological nitrogen fixation (BNF) using natural-abundance ¹^5^N methods, controlled ¹^5^N_2_ - enrichment experiments, and acetylene reduction assays at key growth stages. Exploring *M. symbioticum’s* synergistic potential with resource-conserving agronomic methods, such as low tillage and deficit irrigation, across different maize genotypes may yield substantial increases in sustainability and productivity. Finally, incorporating these agronomic and mechanistic insights into complete economic frameworks—encompassing many trials, varied input costs, and market premiums for sustainably produced grain—will be useful in turning experimental discoveries into scalable, policy-relevant solutions.

## 5 Conclusions

This study demonstrates the potential role of *M. symbioticum* as a foliar-applied microbial biostimulant in sustainable maize production. The bacterial treatment can successfully colonize maize leaves and positively influence vegetative growth, maintain higher chlorophyll content by delaying leaf senescence, foster root development, grain yield and nutritional value. The observed morphological improvements highlight the complex physiological interplay between bacterial endophytes and maize metabolism. Notably, the bacterium’s ACC deaminase activity most likely leads to delayed leaf senescence, which maintains photosynthetic capacity throughout crucial development stages. Moderate N levels paired with bacterial inoculation have the potential to increase grain yield and protein content, suggesting that chemical N inputs can be partially reduced without compromising crop performance, contributing simultaneously to reduce water pollution and greenhouse gas emissions, although these differences were not consistently statistically significant, emphasizing the importance of caution in interpretation.

The foliar biofertilization approach described here is novel and holds promise as a complementary strategy to reduce synthetic nitrogen inputs, although environmental conditions and application methods will likely affect effectiveness. These findings support further research into site-specific agronomic adjustments and optimizing formulation stability for plant inoculation, application protocols, and large-scale field trials to confirm agronomic benefits and economic viability. Collectively, these findings indicate that the incorporation of *M. symbioticum* presents a promising strategy to enhance maize resilience and sustainability under a changing climate scenario.

## Data Availability

The raw data supporting the conclusions of this article will be made available by the authors, without undue reservation.
