# Supplementary material for: Effects of foliar-sprayed bio-fertilizer with N-fixing Methylobacterium symbioticum on morpho-physiological traits of maize under varying N fertilization rates
Source: Front Plant Sci. 2025 Sep 4;16:1661290. doi: 10.3389/fpls.2025.1661290 (PMC12444665; doi:10.3389/fpls.2025.1661290)
Supplement: Supplementary file 1 [file DataSheet1.docx]

Supplementary Material

# Supplementary Figures

**Figure S1.** Dynamics of daily average temperature (line chart, left Y) and precipitation (vertical bars, right Y) at Legnago-Vangadizza (Verona, IT) during the April-August 2023 maize season, highlighting key agricultural practices (dotted lines), such as sowing, chemical dress N fertilization, and foliar spraying of *Methylobacterium symbioticum*.


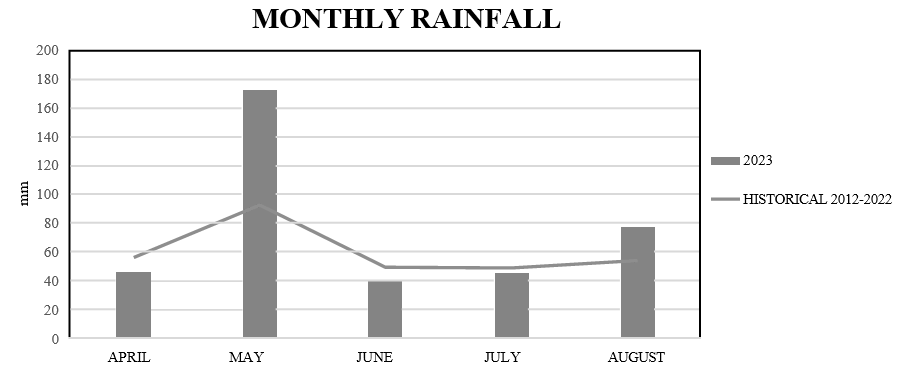


**Figure S2.** Monthly cumulative rainfall trend in Vangadizza (Legnago, Verona – NE Italy) during the experimental trial for the trial period (April – August 2023). The data is compared to the monthly average of the decade from 2012 to 2022.


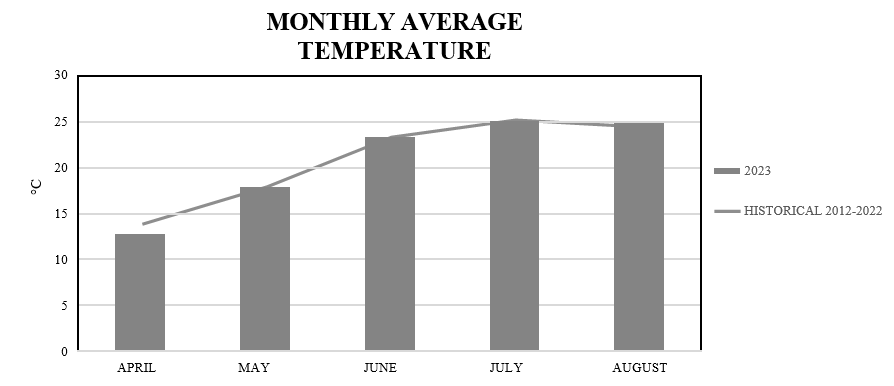


**Figure S3.** Monthly average temperature trends in Legnago-Vangadizza (Verona, IT) during the experimental trial for the trial period (April 2023 – August 2023). Data are compared to the monthly average of the decade from 2012 to 2022.

# Supplementary Tables

**Table S1.** Nitrogen accumulation (mean ± se; kg ha⁻¹) in grains, crop residues, and total for maize under varying treatments (N300, N300 + MS, N350, and N320 + MS). Percentage variation (%Var/C) is calculated relative to the control treatment (N300, Ref.). Within the same parameter, different letters indicate statistically significant differences among treatments (Tukey’s test, p ≤ 0.05) .

| Treatment | Grain N  (kg ha⁻¹) (mean ± se) | %Var/C | |  | Crop residues N  (kg ha⁻¹) (mean ± se) | %Var/C | |  | Total N  (kg ha⁻¹) (mean ± se) | %Var/C |  |
| --- | --- | --- | --- | --- | --- | --- | --- | --- | --- | --- | --- |
| N300 | 150 ± 6.06 | Ref. | b | | 85 ± 4.7 | Ref. | bc | | 235 ± 2.4 | Ref. | c |
| N300 + MS | 176 ± 1.50 | +17% | a | | 129 ± 7.0 | +51% | a | | 305 ± 6.3 | +30% | a |
| N350 | 114 ± 3.63 | –24% | c | | 73 ± 2.7 | –14% | c | | 187 ± 4.9 | –20% | d |
| N320 + MS | 160 ± 3.77 | +6% | ab | | 117 ± 7.5 | +37% | ab | | 277 ± 5.0 | +17% | b |
